# Hippocampal Mitochondrial Dysfunction and Synaptic Disruption Link Organophosphate Exposure to Pre-Diabetes: An LC-MS/MS-Based Proteomics Approach

**DOI:** 10.3390/biom16070952

**Published:** 2026-06-26

**Authors:** Vishal Sandilya, Rowan E. Arida, Sherifdeen Onigbinde, Sarah Sahioun, Favour Chukwubueze, Hadi Al Sheikh, Heba-Tallah Abd Elrahim Abd Elkader, Salwa A. Abuiessa, Mahmoud Agami, Mai M. Helmy, Ahmed El-Yazbi, Yehia Mechref

**Affiliations:** 1 Department of Chemistry and Biochemistry, Texas Tech University, Lubbock, TX 79409, USA; vishal.sandilya@ttu.edu (V.S.); sonigbin@ttu.edu (S.O.); ssahioun@ttu.edu (S.S.); fchukwub@ttu.edu (F.C.); halsheik@ttu.edu (H.A.S.); 2 Department of Pharmacology and Toxicology, Faculty of Pharmacy, Alexandria University, Alexandria 21521, Egypt; ressam@aiu.edu.eg (R.E.A.); salwa.aborajeh@alexu.edu.eg (S.A.A.); mai.helmy@alexu.edu.eg (M.M.H.); 3 Faculty of Pharmacy and Research & Innovation Hub, Alamein International University, Alamein 51718, Egypt; mahmoud.a.agami@gmail.com; 4 Zoology, Biological, and Geological Sciences Department, Faculty of Education, Alexandria University, Alexandria 21526, Egypt; hebatallah@alexu.edu.eg

**Keywords:** organophosphate exposure, LC-MS/MS, proteomics, pre-diabetes, arachidonic acid, cognitive impairment, hippocampus, mitochondrial dysfunction, chlorpyrifos

## Abstract

Organophosphate pesticides (OPPs) are widely used in agriculture and are associated with metabolic dysregulation and cognitive impairment. Emerging evidence links OPP exposure to insulin resistance and diabetes mellitus, a condition known to negatively impact brain function. Prior investigations in our laboratory identified significant dysregulation of arachidonic acid (AA) metabolites associated with the endocannabinoid system in both diabetic patients and those with chronic OPP exposure, with a marked reduction in serum AA levels in the OPP-exposed cohort. This study investigates the impact of OPP exposure and pre-diabetes on the hippocampal proteome and whether AA supplementation can mitigate the resulting neuronal proteomic alterations. Using a controlled rat model, high-resolution LC-MS/MS-based proteomics identified differentially expressed proteins across experimental groups. Both OPP exposure and pre-diabetes were associated with increased cognitive impairment and were associated with overlapping disruptions in pathways related to insulin resistance, mitochondrial function, synaptic plasticity, and neuronal development. AA supplementation mitigated cognitive decline and stabilized synaptic and metabolic proteins; however, residual pathway dysregulation highlights the complexity of these stressors. Our results reveal novel molecular intersections between environmental and metabolic drivers of cognitive impairment, establishing a rationale for further research into inexpensive, protective dietary interventions.

## 1. Introduction

Organophosphate pesticides (OPPs) remain one of the most widely used agricultural chemical classes and are prevalent contaminants in environmental and biological matrices [1,2,3,4]. Studies have shown that individuals with long-term occupational or environmental exposure to OPPs exhibit elevated fasting insulin and glucose levels, impaired glucose tolerance, and a higher incidence of metabolic syndrome [1,2,3,4]. These effects are attributed to the ability of OPPs to disrupt pancreatic β-cell function, alter hepatic gluconeogenesis, and impair insulin signaling pathways [4,5,6,7,8,9]. Mechanistically, OPPs such as chlorpyrifos, malathion, and parathion induce oxidative stress and inflammatory cytokine production (e.g., TNF-α, IL-6), which interfere with insulin receptor substrate (IRS) phosphorylation and downstream Akt activation, leading to decreased glucose uptake in peripheral tissues [10,11,12]. Moreover, OPP-induced mitochondrial dysfunction and endoplasmic reticulum stress further contribute to β-cell apoptosis and reduced insulin secretion capacity. Animal models corroborate these findings, showing that chronic low-dose OPP exposure produces hyperinsulinemia, glucose intolerance, and adipose tissue inflammation [13,14,15]. Collectively, these data suggest that organophosphate exposure may contribute to diabetes pathogenesis by disrupting endocrine and inflammatory homeostasis.

Cognitive impairment encompasses a broad range from mild cognitive impairment (MCI) to dementia, with Alzheimer’s disease (AD) being the most prevalent form [16]. Increasing evidence over the past decade suggests a link between brain insulin resistance (BIR) and the onset of cognitive impairment [16,17,18,19]. Furthermore, this link extends to individuals in the prediabetic stage, where blood glucose levels are higher than usual but not yet high enough to be classified as type 2 diabetes mellitus (T2DM). Pre-diabetes, even without overt T2DM, has been associated with an increased risk of cognitive decline [20,21]. Chronic exposure to OPPs has been hypothesized as a risk factor for metabolic disorders, including T2DM, with exposed individuals possessing higher blood insulin levels [3,22].

Significantly, recent examination of serum metabolomics of T2DM and chronic OPP exposure patients demonstrated alterations in arachidonic acid (AA) metabolites linked to the function of the endocannabinoid system, with a reduction in AA serum levels in the latter group [23,24,25]. AA is a 20-carbon ω-6 polyunsaturated fatty acid defined by four cis-bonds at positions 5, 8, 11, and 14 [26]. As a critical structural component of the cellular phospholipid bilayer, AA is primarily sequestered from dietary animal sources to maintain membrane integrity and signaling precursors [27,28]. AA serves as a fundamental mediator of cellular signaling, neurophysiology, and immune homeostasis. It is the primary biosynthetic precursor for a diverse array of bioactive eicosanoids, including prostaglandins, leukotrienes, thromboxanes, and epoxyeicosatrienoic acids (EETs) [29]. Furthermore, AA serves as a critical modulator of the endocannabinoid system by acting as a primary biosynthetic precursor for major endocannabinoids, including *N-*arachidonoyl ethanolamine (anandamide) and 2-arachidonoylglycerol (2-AG) [30]. Beyond its role as a precursor, AA regulates cellular homeostasis through three primary mechanisms: modification of membrane organization and lipid rafts, modulation of secondary messengers derived from the membrane, and functioning as a precursor for additional lipid mediators [29]. AA demonstrates a dual role in inflammation; while various AA metabolites, such as those derived from cyclooxygenases, lipoxygenases, and cytochrome P450, are known to exhibit pro-inflammatory actions, other metabolites, such as lipoxins A4 (LXA4) and B4 (LXB4), demonstrate significant anti-inflammatory and pro-resolving effects [31,32,33,34]. Additionally, AA has demonstrated competitive binding with palmitic acid at the MD2 (lymphocyte antigen 96) receptor, an essential extracellular co-receptor for Toll-like receptor 4 (TLR4) [31]. By occupying the hydrophobic pocket of MD2, AA prevents the dimerization of the TLR4 complex without eliciting the pro-inflammatory cytokine production (such as TNF-α and IL-6) typically induced by saturated fatty acids or lipopolysaccharides.

Despite mounting evidence of systemic toxicity, the proteomic effects of OPP exposure within the brain remain insufficiently characterized. We previously analyzed proteomic alterations in human blood serum following acute and chronic exposure to OPPs [35]. Our findings revealed a significant increase in inflammatory markers and a predicted increase in carcinogenesis upon chronic OPP exposure [35]. Notably, prolonged exposure resulted in upregulation of pro-inflammatory proteins such as S100A8 and von Willebrand factor (VWF), indicative of heightened immune activation, along with predicted downregulation of LXR/RXR signaling and DHCR24 pathways, pointing to impaired lipid regulation and increased neurotoxic vulnerability. These findings suggest that chronic organophosphate exposure contributes to a pathological state characterized by inflammation-driven neurodegeneration and increased long-term health risks, including cognitive impairment.

This study used a controlled rat model to explore the potential of AA supplementation to modulate OPP-induced hippocampal proteomic dysregulation under both non-diabetic and pre-diabetic metabolic conditions. We also examined proteomic alterations following OPP exposure and their comparison with changes observed in pre-diabetic rats with respect to insulin resistance. Given the hippocampus’s critical role in learning and memory and its susceptibility to insulin resistance and neuroinflammation, our investigation employed high-resolution LC-MS/MS proteomics to elucidate the biochemical pathways underlying OPP-induced neurotoxicity. Furthermore, we assessed whether AA supplementation confers neuroprotective effects by restoring synaptic, metabolic, and inflammatory functions. To our knowledge, this represents the first proteomics-based study to evaluate AA as a therapeutic modulator of OPP-induced cognitive deficits in the context of impaired glucose regulation, thereby addressing a significant gap in understanding the interplay between environmental toxicants, metabolic disorders, and cognitive decline.

## 2. Materials and Methods

### 2.1. Chemicals and Reagents

HPLC-grade water, acetonitrile, and MS-grade formic acid were all sourced from Fisher Scientific. Ammonium bicarbonate, dithiothreitol, and iodoacetamide were obtained from Sigma-Aldrich. Promega supplied the MS-grade Trypsin/Lys-C mix for protein digestion.

### 2.2. Animal Model

This study was conducted according to a protocol approved by the Ethics Committee for the Use and Care of Laboratory Animals established by the Faculty of Pharmacy, Alexandria University (No. AU 0620238131191), on 13 August 2023. Pilot experiments were performed to select the exposure dose that allows for chronic exposure without rapid deterioration. Additionally, dermal exposure measures were taken to avoid acute oral toxicity following CPY exposure. Novel object recognition tests were used as the primary outcome for determining the appropriate sample size. Sixty 4–6-week-old male Sprague-Dawley rats weighing 150–220 g were randomized into the six groups highlighted in Table 1, with each animal representing a single experimental unit. Rats were obtained from the Animal Care Unit, Experimental Animal Center in Alamein International University, and maintained on a standard laboratory diet and water *ad libitum*. Animals were housed in a well-ventilated, temperature-controlled room (23 ± 2 °C, 55% relative humidity) and exposed to 12 h dark/light cycles. Three animals were housed per cage. The study was performed in an open-label manner.

Prediabetes (PD) was induced in 30 rats by twelve weeks of a mild hypercaloric diet feeding as described in our previous research, producing a phenotype characterized by normal weight, euglycemia, hyperinsulinemia, and increased body fat composition and insulin resistance [36,37]. Standard chow (Al-Eman Foundations Group, Alexandria, Egypt) had a caloric composition of 21% protein, 5.9% crude fat, and 3% crude fiber, with an energy density of 3 kcal/g. The high-fat diet was prepared by weight from standard chow (65%), fructose (20% *w*/*w*; Specialized Food Industry Co., 10th of Ramadan City, Egypt), and saturated fat (15% *w*/*w*; Afia International Company, Cairo, Egypt). Body weights were recorded weekly so that daily food allotments could be scaled to ensure appropriate caloric intake. Control (CTRL) animals received 12.5 g of food per 100 g body weight, whereas prediabetic (PD) animals received 7.5 g per 100 g body weight. This restricted-feeding scheme was chosen to induce metabolic dysfunction while avoiding an excess caloric load. Daily intake was quantified by weighing the food provided and subtracting the amount remaining the following day; these values were then summed to calculate weekly caloric intake.

At the end of the twelve weeks, 20 control and 20 PD rats were dermally exposed to chlorpyrifos (CPY, Central Agricultural Pesticides Lab, Agricultural Research Center, Cairo, Egypt) via their tails, using a surgical pad soaked with CPY diluted in DMSO (Alpha Chemika Company, Andheri, India) as described previously [38,39]. A CPY dose of 80.8 mg/kg, corresponding to 40% of the dermal LD_50_, was administered [36]. The exposure lasted 5 h per day every other day for the exposure duration of 35 days. To minimize acute oral exposure, animals were housed individually for the duration of treatment with the surgical pad secured with a surgical silk tape. Control animals were exposed to plain DMSO-soaked pads. Half of the CPY-exposed rats in either group received 3 mg/kg AA once daily by oral gavage, starting on the 13th week. AA was dissolved in olive oil, which was used as the vehicle control for untreated rats. The selected AA dose has previously been shown to reduce insulin resistance in rats fed a high-fat diet [40].

At the conclusion of the study, fasting blood glucose was assessed using a colorimetric assay kit (Cat. No. 250-001, Spectrum Diagnostics, Obour, Egypt). Serum insulin concentrations were quantified with a rat insulin enzyme-linked immunosorbent assay (ELISA) kit (Cat. No. 80-INSHU-E01.1, E10.1, ALPCO Diagnostics, Salem, NH, USA), and insulin resistance was calculated using the homeostatic model assessment of insulin resistance (HOMA-IR).

### 2.3. Cognitive Testing

Hippocampal-dependent cognitive performance was assessed using the novel object recognition (NOR) and the Y-maze tests. The NOR test was used to assess the rat’s ability to distinguish between familiar and novel objects as described previously [41]. This test is often used to evaluate short-term memory by adjusting the retention interval. Conducted in a single day, the experiment included two phases: a training phase and a test phase, each lasting three minutes, with a thirty-minute rest period in between, and a novelty discrimination index (DI) was calculated. The Y-Maze was used to assess spatial learning and memory. It consisted of three arms: “A” (home arm), “B” (familiar arm), and “C” (novel arm). The test spanned three consecutive days. During the first two days, rats underwent 10 min training sessions with only arms “A” and “B” open, while arm “C” remained closed. On the third day, the test lasted for 5 min with all three arms open. The percentage of spontaneous alternation performance (SAP%) was calculated as described previously [42,43].

### 2.4. Tissue Homogenization and Protein Digestion

Five animals from each cohort, with the exception of PD + CPY + AA, were utilized for LC-MS/MS based proteomic analysis, while the rest were utilized for behavioral analyses. One animal in the Prediabetes + CPY + AA group was lost during isoflurane anesthesia prior to exsanguination and tissue collection. Consequently, the final sample size for this group was reduced by one compared with the other experimental groups (Table 1).

At the end of the experiment protocols, rats were euthanized by exsanguination under isoflurane anesthesia. The scalp was incised, the skull was exposed, and it was carefully drilled. The brains were quickly removed, weighed, washed with ice-cold normal saline, and dissected to isolate the cortical and hippocampal areas. Half of the dissected hippocampal tissues were flash-frozen and stored at −80 °C. Approximately 50 mg of hippocampus tissue was thawed at room temperature and resuspended in 2.5% sodium deoxycholate (SDC) in 50 mM ammonium bicarbonate buffer (pH = 7.5) along with 400 µm molecular biology-grade zirconium beads (BMBZ 400-250-36, OPS diagnostics, Lebanon, NJ, USA) in a 2 mL microtube. The tissue was then homogenized using a bead beater (Beadbug microtube homogenizer, Benchmark Scientific, Sayreville, NJ, USA) at 4 °C to prevent overheating and minimize endogenous protease activity. The bead beater was operated at 4K revolutions/min for 30 s, five times, with 30 s pauses for cooling. The homogenized tissue was then sonicated on ice for one hour to facilitate protein dissolution. Following sonication, samples were centrifuged at 21,000× *g* for 10 min, and the supernatant was extracted and stored at −80 °C prior to subsequent analysis.

Following homogenization, formic acid was added to the sample at 1% of the total volume to precipitate SDC. The samples were subsequently centrifuged at 21,000× *g* for 10 min, and the supernatant was collected for further analysis. An equivalent of 50 µg of protein was then taken from each sample and denatured at 85 °C for 15 min. The denatured proteins were then reduced by adding 1.25 µL of 200 mM dithiothreitol (DTT) and incubating at 60 °C for 45 min. The reduced samples were then carbamidomethylated using 5 µL of 200 mM iodoacetamide (IAA) and incubated at 37 °C for 30 min. The excess IAA was then quenched via a second addition of 1.25 µL of 200 mM DTT and incubation at 37 °C for 30 min. Lastly, trypsin was added to the sample at a 1:25 enzyme-to-protein ratio (2 µg trypsin), followed by incubation at 37 °C for 18 h. Following tryptic digestion, 0.5 µL of formic acid was added to quench trypsin, and the samples were subsequently dried.

### 2.5. C-18 Desalting

Samples were desalted using C18 Top-Tip columns according to the following protocol. First, releasing solution 1 (RS1: 60% acetonitrile, 39.9% HPLC H_2_O, 0.1% formic acid), releasing solution 2 (RS2: 100% acetonitrile), and binding solution (BS: 0.1% formic acid in HPLC H_2_O) were prepared. Samples were dissolved in 50 µL of binding solution, and the Top-Tip C18 columns were washed with 50 µL RS1 three times (1000× *g*, 1 min) followed by conditioning with 50 µL BS three times (1000× *g*, 1 min). Samples were then loaded onto the tip and centrifuged at 500× *g* for 2 min until completely loaded into the C18 bed, followed by a 2 min incubation. The pass-through was centrifuged at 500× *g* for 1–2 min and reloaded onto the tip. After washing with 50 µL BS three times (1000× *g*, 1 min), the collecting tube was changed, and samples were eluted with 50 µL RS1 three times (1000× *g*, 1 min) followed by elution with 50 µL RS2 three times (1000× *g*, 1 min). The collected samples were then dried and resuspended in 2% ACN and 0.1% formic acid.

### 2.6. LC-DDA-MS/MS Proteomic Analysis

**Figure 1:**
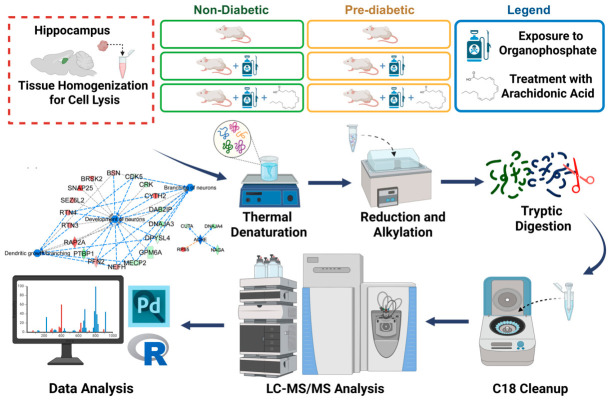
Summary of the experimental workflow highlighting sample preparation and instrumental analysis steps. Created in BioRender. Group, M. (2026) https://BioRender.com/tqnfaib (accessed on 15 June 2026).

For LC-MS/MS analysis, 1 µL of each sample, corresponding to 1 µg of total protein, was injected onto a C18 trap column (75 µm × 2 cm, 2 µm particle size, 100 Å pore size; Thermo Scientific, Pittsburg, PA, USA) and retained for 10 min. The samples were then transferred to an Acclaim PepMap C18 nano column (75 µm × 15 cm, 2 µm, 100 Å; Thermo Scientific, Pittsburgh, PA, USA) using an Ultimate 3000 nanoUHPLC system (Dionex, Sunnyvale, CA, USA) operating at a flow rate of 300 nL/min and a column temperature of 29.5 °C. Mobile phase A (MPA) consisted of an aqueous solution containing 2% acetonitrile (ACN) and 0.1% formic acid (FA), while mobile phase B (MPB) was composed of 99.9% ACN and 0.1% FA. The peptide separation used a time-programmed gradient with mobile phase B concentrations as follows: the system began with 5% MPB and maintained this concentration for the first 10 min. The concentration then increased linearly to 35% over 11–80 min, followed by a further increase to 60% between 80 and 110 min. Then MPB was rapidly increased to 90% between 110 and 113 min and held steady for 5 min. The system then quickly returned to a 5% MPB over 1 min and remained at 5% for the final equilibration period from 119 to 120 min. The nanoUHPLC system was coupled to a Thermo Scientific Orbitrap QExactive HF mass spectrometer (San Jose, CA, USA) configured for positive ion detection. Data collection employed a data-dependent acquisition approach, with the primary mass spectrum (MS1) acquired at a resolution of 120,000 across an *m*/*z* range of 400–1800, using an AGC target of 1 × 10^6^. Fragment ion spectra (MS/MS) were collected at 30,000 resolution, with a 60 s dynamic exclusion period to prevent reanalysis of the same peptide. Fragmentation was performed using assisted-collision-energy HCD (HCD) with normalized collision energies of 15, 25, and 35. The complete proteomic experimental design is illustrated in Figure 1.

### 2.7. LC-PRM-MS/MS Validation

For LC-PRM-MS/MS validation, 1 µL of each sample, corresponding to 1 µg of total protein, was utilized. The LC parameters mimicked those used in LC-DDA-MS/MS analysis. The nano UHPLC was coupled to a Thermo Scientific Lumos Tribrid mass spectrometer (San Jose, CA, USA) configured for positive ion detection. Data collection was performed in targeted MS^2^ mode at a resolution of 30,000, with an *m*/*z* isolation window of 1.6. The AGC target was set to auto, and the scan range was set to standard. Fragmentation was performed using assisted-collision-energy HCD with normalized collision energies of 15, 25, and 35.

### 2.8. Data Analysis

Protein identification and quantification were conducted using Proteome Discoverer 3.2 (Thermo Scientific) with searches performed against the UniProtKB Rattus Norvegicus reference proteome database (Accessed April 2025) using the SequestHT search engine. Search parameters were configured to accept peptides ranging from 5 to 60 amino acids in length with a precursor mass tolerance of 10 ppm and a fragment mass tolerance of 0.02 Da. Carbamidomethylation of cysteine was set as a fixed modification, while protein N-terminus acetylation and methionine oxidation were designated as variable modifications. Identification criteria included 1% FDR for classification as high confidence.

**Figure 2:**
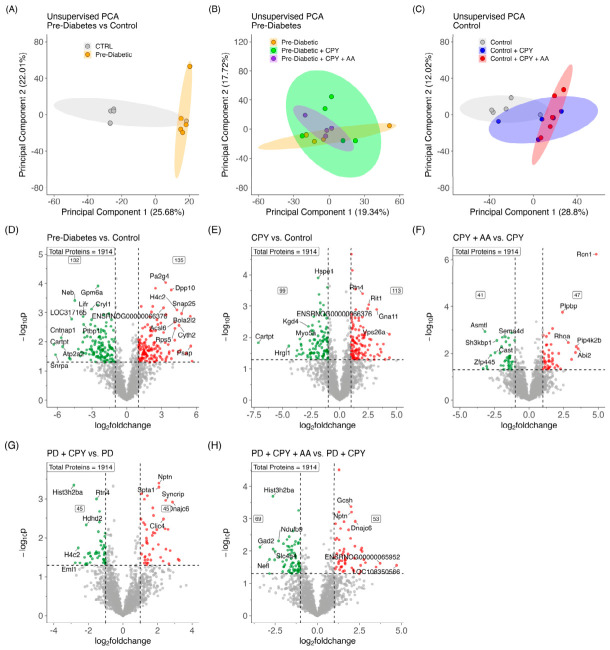
Unsupervised principal component analysis (PCA) plot of the entire hippocampal proteome for (**A**) CTRL vs. Pre-diabetes, (**B**) Pre-diabetic cohort comparison, and (**C**) Non-diabetic cohort comparison. Volcano plots displaying the significantly altered proteins in (**D**) Pre-diabetes vs. control (PD vs. CTRL), (**E**) chlorpyrifos exposure vs. control (CPY vs. CTRL), (**F**) AA-treated chlorpyrifos-exposed vs. control (CPY + AA vs. CTRL), (**G**) chlorpyrifos-exposed pre-diabetes vs. pre-diabetes (PD + CPY vs. PD), and (**H**) chlorpyrifos-exposed and AA-supplemented pre-diabetes vs. pre-diabetes (PD + CPY + AA vs. PD).

The identified proteins were filtered to retain only those with high confidence (FDR < 1%) and those quantified in >70% of the sample. After filtering, the proteins were log_2_- and quantile-normalized in R using the preprocessCore package (v1.74.0). Following filtering and normalization, unsupervised principal component analysis (PCA) was used to visualize the separation of the identified proteome from hippocampal tissue across different cohorts. The principal components were ranked by variance explained and displayed in a 2-dimensional scatter plot, as shown in Figure 2. To complement the unsupervised PCA with a formal test of group separation, permutational multivariate analysis of variance (PERMANOVA) was performed on the normalized values using the adonis2 function in the vegan R package (v2.7-5). Pairwise Euclidean distances between samples were computed so that the test operated in the same space as the PCA, and the proportion of multivariate variance attributable to the experimental group was evaluated against 9999 free permutations of the group labels. An omnibus model across all six groups was fitted first, followed by pairwise comparisons between groups. Homogeneity of multivariate dispersion among groups was assessed with the betadisper routine to verify that significant PERMANOVA outcomes reflected differences in group centroids rather than differences in within-group spread. Significance was set at *p* < 0.05.

Prior to differential expression analysis, the normality of protein abundance distributions was assessed using the Shapiro–Wilk test applied to each protein across all experimental groups. Over 90% of proteins exhibited normally distributed abundance values (*p* > 0.05), supporting the use of a parametric testing framework. Consequently, a pairwise Welch’s *t*-test was performed to identify differentially expressed proteins (DEPs), with a *p*-value < 0.05 and |log2FC| > 1 (indicating a two-fold increase or decrease) serving as the cutoff for significance. Given the limited statistical power inherent of animal models and modest effect sizes, nominal *p-*values coupled with an effect-size cutoff (|log2FC| > 1) were used rather than multiple testing correction, as the primary objective of this study was pathway-level enrichment analyses rather than individual biomarker discovery; accordingly, the biological relevance of findings was assessed through downstream enrichment and network analyses rather than reliance on individual protein-level significance. Additionally, all pathway enrichment studies used FDR-corrected *p*-values.

After the comparative statistical analysis, protein–protein interaction, Gene Ontology, and KEGG enrichment analyses were performed using the STRING database (accessed 17 April 2025, https://string-db.org/). The entire string network, encompassing both functional and physical protein associations, was employed. Proteins were subsequently filtered to retain only those that exhibited two or more interactions with an interaction score exceeding 0.4. Furthermore, the analysis exclusively focused on interactions involving the query proteins, excluding any additional interactors. Additionally, Reactome and KEGG enrichment analyses were performed using the DAVID functional annotation tool by NIH (Accessed 17 April 2025), and the results were visualized using the circlize (v0.4.18) and ggplot2 (v4.0.3) packages in R (v4.4.2).

Ingenuity Pathway Analysis (IPA) by Qiagen was employed to identify significantly activated or inhibited canonical pathways, diseases, and biological functions [44]. The Uniport accession, *p*-value, and log2FC values for the filtered DEPs were uploaded to IPA, followed by an enrichment analysis using the log2FC values. The enriched canonical pathways were filtered only to retain those with |z-score| greater than 1.5 and an adjusted *p*-value less than 0.05. Furthermore, IPA was used to perform a comparative analysis of each comparison to identify the commonly altered canonical pathways across cohorts. Additional figures, including volcano plots summarizing differential expression profiles, Venn diagrams illustrating the overlap of DEPs between different comparisons, and bar plots, were generated in R (v4.5.0) using the ggplot2 (v4.0.3) and ggvenn (v0.1.19) packages [45]. The ComplexHeatmap package (v2.13.1) in R was used to create hierarchically clustered heatmaps and IPA common pathway heatmaps [46].

## 3. Results

### 3.1. Insulin Resistance and Behavioral Measures

Fasting glucose was significantly elevated in CPY-exposed rats relative to controls, and this elevation was attenuated by AA treatment, which produced a significant decrease compared with the CPY group (Table 2). PD rats showed a non-significant increase in blood glucose relative to controls; however, CPY exposure in PD rats again significantly raised glucose compared with PD alone, with AA treatment producing a comparable reduction (Table 2).

For the discrimination index, control animals showed robust object discrimination, whereas DI was significantly reduced in both the CPY and PD groups relative to control. AA treatment restored performance, with the CPY + AA group differing significantly from CPY and the PD + CPY + AA group differing significantly from PD + CPY. A comparable pattern was observed for spontaneous alteration performance. SAP% was markedly lower in CPY animals than in CTRL, while CPY + AA animals performed significantly better than CPY. The PD, PD + CPY, and PD + CPY + AA groups showed intermediate-to-recovered alteration behavior, though these differences did not reach statistical significance under the post hoc comparisons reported.

### 3.2. Protein Identification and Differential Expression Analysis

A total of 1914 proteins were quantified in more than 70% of the samples with high confidence. A clear separation was observed in the unsupervised principal component analysis between control and PD rats (Figure 2A), suggesting a shift in the hippocampal proteome. Unsupervised PCA also displayed a limited separation between control and CPY-exposed rats (Figure 2C); however, there was no clear separation between CPY-exposed PD rats and non-exposed PD rats (Figure 2B). The 2-dimensional unsupervised PCA plots for individual comparisons are presented in Supplementary Figure S1. To statistically corroborate the proteomic differences visualized by PCA, PERMANOVA was performed across all experimental groups. Group membership had a significant effect on the hippocampal proteome and accounted for approximately 32% of the total multivariate variation (pseudo-F = 2.16, R^2^ = 0.32, *p* < 0.001; 9999 permutations, Euclidean distances). Pairwise comparisons corresponding to the PCA panels indicated nominal separation between control and pre-diabetic rats (pseudo-F = 3.06, R^2^ = 0.28, *p* = 0.038) and a comparable, non-significant trend between control and CPY-exposed rats (pseudo-F = 2.47, R^2^ = 0.24, *p* = 0.073). Consistent with the limited overlap seen by PCA, CPY exposure produced no significant proteomic separation within the pre-diabetic cohort. Multivariate dispersion did not differ significantly among groups (PERMDISP, F = 0.43, *p* = 0.84), indicating that the significant PERMANOVA result reflected differences in group centroids rather than heterogeneity of within-group variance. Collectively, these analyses confirm that overall proteomic structure differed significantly among groups, while individual pairwise differences were modest in this pilot-scale cohort and are therefore interpreted as exploratory.

Because the study was powered for pathway-level rather than single-protein inference, differentially expressed proteins were defined by a nominal *p* < 0.05 together with |log_2_FC| > 1 as a discovery screen; Benjamini–Hochberg-adjusted q-values are reported for all proteins (Supplementary Table S1). As expected for a pilot-scale cohort tested across ~1900 proteins, few proteins survived genome-wide FDR correction at the individual level. Inference therefore rests not on single-protein significance but on FDR-corrected pathway enrichment, the concordant directionality of the 97 shared proteins (binomial *p* < 10^−29^), significant multivariate group separation (PERMANOVA, *p* < 0.001), and orthogonal PRM validation of key targets.

Differential expression analysis using Welch’s *t*-test revealed a total of 267 DEPs (*p*-value < 0.05 and |log2foldchange| > 1) between pre-diabetes and control cohorts. Additionally, 212 proteins were differentially expressed between organophosphate-exposed (CPY) vs. control, 88 in organophosphate- and arachidonic acid-treated (CPY + AA) vs. CPY, 90 in OPP-exposed pre-diabetic (PD + CPY) vs. pre-diabetic (PD), and 122 in OPP-exposed and AA-treated pre-diabetic (PD + CPY + AA) vs. PD + CPY. A summary of differential expression analysis results is provided in Table 3 and shown as volcano plots in Figure 2D–H. The expression levels of all DEPs across each comparison, along with their nominal and adjusted *p*-values, are provided in Supplementary Table S1. Re-analysis with limma’s moderated *t*-test recovered proteins surviving genome-wide FDR correction in four of five comparisons: 158 for PD vs. CTRL, 54 for CPY vs. CTRL, 8 for PD + CPY vs. PD, and 2 for PD + CPY + AA vs. PD + CPY (all q < 0.05; Supplementary Table S3), each of which also met the |log_2_FC| > 1 threshold. Only the CPY + AA vs. CPY comparison, the smallest in effect size, yielded no FDR-significant proteins. These results indicate that the principal proteomic differences are robust to multiple-testing correction.

### 3.3. Protein–Protein Interaction, Gene Ontology, and Ingenuity Pathway Analysis

**Figure 3:**
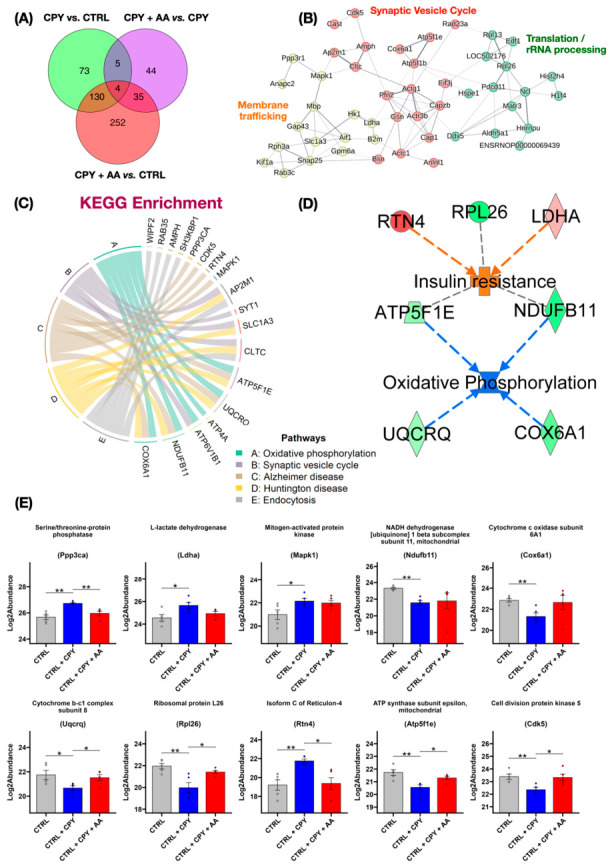
(**A**) Venn diagram showing commonly altered DEPs between CPY vs. CTRL, CPY + AA vs. CPY, and CPY + AA vs. CTRL. (**B**) Protein–protein interaction analysis using the STRING database for DEPs in CPY vs. CTRL highlighting three main cluster of protein interactions involving membrane trafficking, synaptic vesicle cycle, and translation/rRNA processing. (**C**) KEGG enrichment for proteins (**B**) highlighting involvements in neurogenerative diseases such as Alzheimer’s disease and Huntington’s disease and pathways such as synaptic vesicle cycle, oxidative phosphorylation, and endocytosis. (**D**) Ingenuity pathway analysis for DEPs in CPY vs. CTRL highlighting an increase in insulin resistance and a significant decrease in oxidative phosphorylation. Blue and orange highlight predict inhibition and activation, respectively. Blue lines indicate a change in the detected molecule leading to an inhibition of the pathway, while orange lines represent activation of the pathway. Grey lines represent an uncertain direction of change. (**E**) Bar plots displaying the abundance of proteins involved in oxidative phosphorylation, synaptic vesicle cycle, or insulin resistance. * Indicates *p*–value < 0.05, ** indicates *p*–value < 0.01.

Comparative proteomic analysis using Venn diagrams revealed differential protein expression patterns across experimental conditions (Figure 3A). The analysis identified 73 proteins exclusively altered by CPY treatment (CPY vs. CTRL), 44 proteins uniquely modified by AA supplementation in CPY-exposed samples (CPY + AA vs. CPY), and 252 proteins specifically changed in the combined treatment relative to control (CPY + AA vs. CTRL).

Intersection analysis showed that 4 proteins were commonly dysregulated in both CPY vs. CTRL and CPY + AA vs. CPY comparisons, 35 proteins overlapped between CPY + AA vs. CPY and CPY + AA vs. CTRL, while 130 proteins were shared between CPY vs. CTRL and CPY + AA vs. CTRL. Protein–protein interaction analysis of proteins altered by CPY treatment (CPY vs. CTRL) revealed three major clusters: membrane trafficking, synaptic vesicle cycle, and translation/rRNA processing (Figure 3B). Snap25, Rpl26, Actg1, Mbp, and Ncl were identified as hubs, with each possessing greater than five connections.

**Figure 4:**
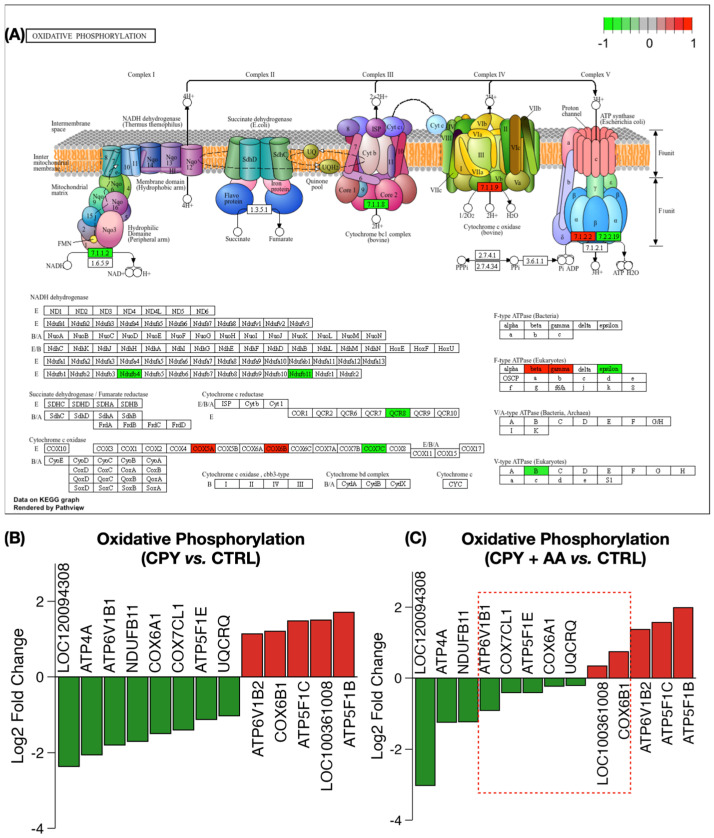
(**A**) Schematic of the electron transport chain showing disruption in complexes 1, 3, 4, and 5. Green boxes indicate downregulated proteins, while red boxes indicate upregulated proteins, and uncolored boxes were either not differentially expressed or were not detected. (**B**) Bar chart showing the log2 of the change in expression of proteins involved in oxidative phosphorylation following chlorpyrifos exposure (CPY vs. CTRL). (**C**) Bar chart showing the expression of the same proteins following AA supplementation (CPY + AA vs. CTRL) with the red box highlighting proteins that were no longer differentially expressed.

KEGG enrichment analysis on these proteins revealed significant alterations in oxidative phosphorylation, synaptic vesicle cycle, and endocytosis (Figure 3C). Additionally, proteins involved in Huntington’s disease and Alzheimer’s disease were also particularly enriched. Ingenuity pathway analysis predicted increased insulin-resistance signaling, inferred by the simultaneous upregulation of Ldha and Rtn4 and the downregulation of Rpl26, Atp5f1e, and Ndufb11. IPA further substantiated the dysregulation in oxidative phosphorylation (Figure 3D). KEGG enrichment showed disruption across complexes I, III, IV, and V of the electron transport chain (Figure 4A). The log2(fold-change) of DEPs in CPY exposure involved in oxidative phosphorylation is shown in Figure 4B. It was noted that the expression of most of these proteins was attenuated and no longer significantly altered following AA supplementation (Figure 4C).

**Figure 5:**
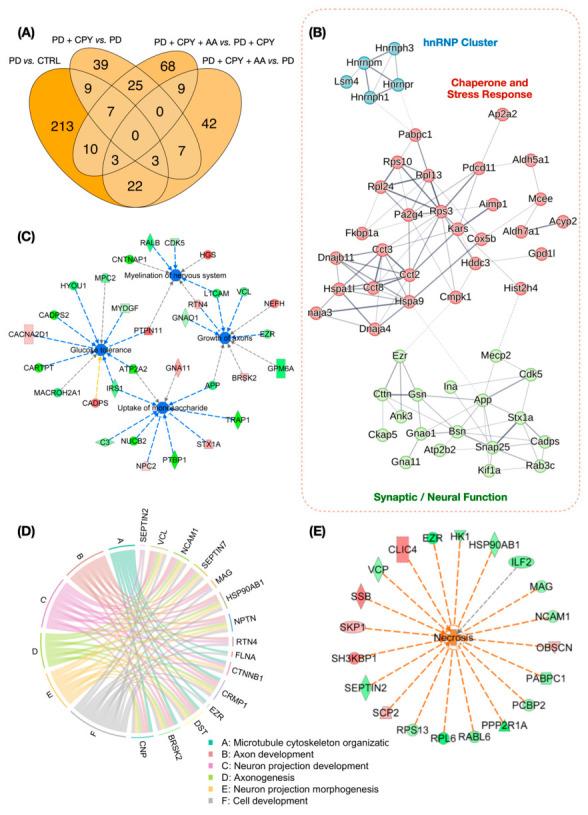
(**A**) Venn diagram displaying commonly altered DEPs between PD vs. CTRL, PD + CPY vs. PD, PD + CPY + AA vs. PD + CPY, and PD + CPY + AA vs. PD. (**B**) Protein–protein interaction analysis using the STRING database for DEPs in PD vs. CTRL displaying three main clusters of proteins including hnRNP proteins, chaperone and stress response-related proteins, and synaptic/neural function-related proteins. (**C**) Ingenuity pathway analysis predicted inhibition (blue) of myelination of nervous system, growth of axons, uptake of monosaccharide, and glucose tolerance in pre-diabetes compared to control (PD vs. CTRL). Green and red represent observed decrease and increase, respectively. (**D**) Chord diagram showing the involvement of key proteins in altered biological processes in pre-diabetes (PD vs. CTRL). (**E**) Predicted increase (orange) in necrosis following exposure of pre-diabetic rats to chlorpyrifos (PD + CPY vs. PD). Blue and orange highlight predict inhibition and activation, respectively. Blue lines indicate a change in the detected molecule leading to an inhibition of the pathway, while orange lines represent activation of the pathway. Grey lines represent an uncertain direction of change.

In the pre-diabetic cohort, Venn diagram analysis identified distinct alterations across treatment groups: 213 proteins were uniquely altered in pre-diabetes relative to controls (PD vs. CTRL), 39 were specifically changed by CPY treatment (PD + CPY vs. PD), 68 were unique to AA supplementation after CPY exposure (PD + CPY + AA vs. PD + CPY), and 42 were unique to the comparison of AA supplementation versus pre-diabetes alone (PD + CPY + AA vs. PD) (Figure 5A). Protein–protein interaction analysis revealed three principal clusters encompassing hnRNP proteins, chaperone and stress response proteins, and synaptic/neural function proteins (Figure 5B). Snap25 was once again identified as a hub protein via PPI analysis, alongside Cct3, Cct8, Cct2, Hspa9, Rps3, Bsn, and Rpl13. IPA demonstrated significant inhibition of myelination of the nervous system, axonal growth, monosaccharide uptake, and glucose tolerance in the pre-diabetic group compared to controls (Figure 5C).

Biological process enrichment revealed a substantial alteration in proteins involved in axon development, microtubule cytoskeleton organization, neuron projection development, axonogenesis, neuron projection morphogenesis, and cell development (Figure 5D and Supplementary Figure S3A). These perturbations in axonal growth and neurogenesis may contribute to the observed cognitive deficits in pre-diabetes relative to controls.

AA supplementation proved effective in reversing the disruption in various proteins involved in neuronal function such as Septin7, Dlg1, Ctnnb1, Nptn, Tanc2, Ezr, Tln2, Flna, Pfn1, and Erc2 (Supplementary Figure S3C). Exposure of pre-diabetic rats to chlorpyrifos showed no further disruption in neuronal function, likely due to the cognitive impairment already being present in pre-diabetic rats (Table 2). However, a significant activation of necrosis was predicted following chlorpyrifos exposure in pre-diabetic rats, suggesting additional worsening effects of organophosphates beyond the risk of developing metabolic disorders (Figure 5E). The expression of proteins involved in the pathways mentioned above is shown in Supplementary Figure S4. The statistical summary of common DEPs with their nominal and adjusted *p*-values is summarized in Supplementary Table S2.

### 3.4. Comparative Analysis Between Organophosphate Exposure and Pre-Diabetes

**Figure 6:**
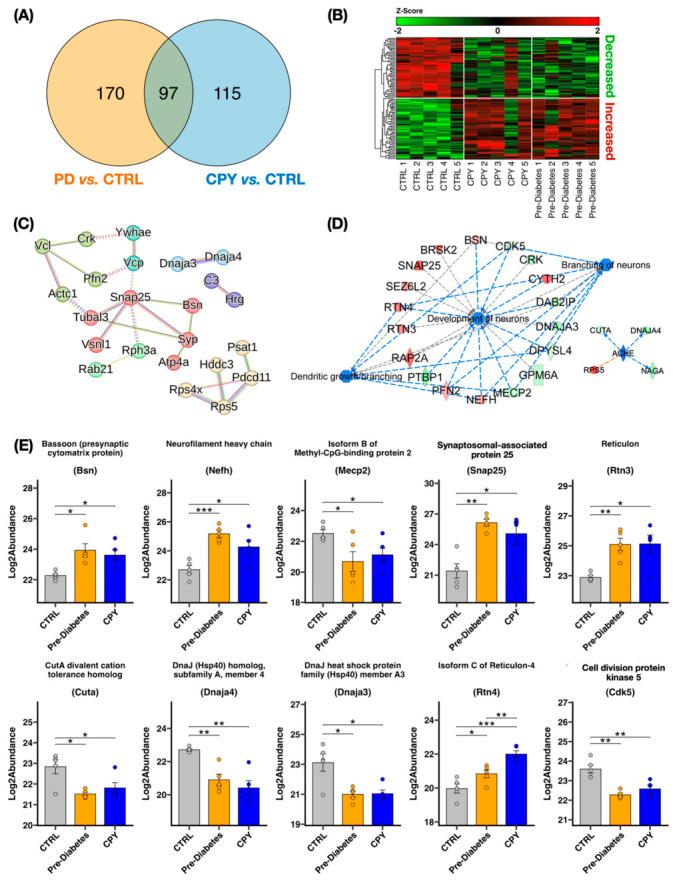
(**A**) Venn diagram highlighting commonly altered proteins between pre-diabetes (PD vs. CTRL) and chlorpyrifos exposure (CPY vs. CTRL). A total of 97 proteins were commonly altered between the two conditions, while 170 were unique to pre-diabetes, and 115 were unique to chlorpyrifos exposure. (**B**) Hierarchically clustered heatmap displaying the expression of the 97 proteins commonly altered between pre-diabetes and chlorpyrifos exposure. Blue and orange highlight predict inhibition and activation, respectively. Blue lines indicate a change in the detected molecule leading to an inhibition of the pathway, while orange lines represent activation of the pathway. Grey lines represent an uncertain direction of change. (**C**) Protein–protein interaction analysis using the STRING database for the 97 commonly altered proteins. (**D**) IPA of the 97 commonly altered proteins predicted inhibition of neuronal development and branching, dendritic growth and branching, as well as ACHE activity. (**E**) Representative boxplots for proteins commonly altered in CPY exposure and Pre-diabetes.* Indicates *p*–value < 0.05, ** indicates *p*–value < 0.01, and *** indicates *p*–value < 0.001.

Comparison analysis between chlorpyrifos-exposed (CPY vs. CTRL) and pre-diabetic (PD vs. CTRL) groups revealed 97 common DEPs (Figure 6A). Interestingly, all 97 DEPs changed in the same direction across both conditions, highlighting the similarity between OPP exposure and prediabetes (Figure 6B,E). The probability of observing concordant directionality across all 97 commonly altered proteins under the null hypothesis of independent perturbations is vanishingly small (binomial test, *p* < 1 × 10^−29^), formally supporting the interpretation that CPY exposure and pre-diabetes converge on shared rather than coincident molecular mechanisms.

PPI analysis of these 97 proteins again showed Snap25 as a hub in the interaction network, with interactions with Bsn, Syp, Tubal3, Vsnl1, and Vcp (Figure 6C). Ingenuity pathway analysis of the same proteins showed significant inhibition of neuronal development and branching (Figure 6D). Additionally, Ache was predicted to be inhibited in both conditions, as demonstrated by decreases in Cuta, Dnaja4, and Naga and an increase in Rps5. The expression of some key proteins involved in the pathways mentioned above is displayed in Figure 6E.

### 3.5. PRM Analysis

Ten proteins were selected for parallel reaction monitoring (PRM) validation based on their involvement in key dysregulated pathways, such as insulin resistance, oxidative stress, mitochondrial function, oxidative phosphorylation, and synaptic function. These proteins included RTN4, GSN, NCAM1, LDHA, PRPH, SOD2, YWHAE, PPP3CA, SKP1, and SNAP25.

Of these, six—RTN4, GSN, NCAM1, LDHA, YWHAE, and SNAP25—were significantly validated, while the remaining proteins showed the same direction of change (increase or decrease) as the LC-DDA-MS/MS analysis without reaching statistical significance (Supplementary Figures S5 and S6).

## 4. Discussion

Significant scientific evidence demonstrates an association between organophosphate pesticide (OPP) exposure and the development of pre-diabetes and eventually diabetes mellitus. This link is supported by substantial epidemiological studies [47], animal research [8], and mechanistic investigations. Several connections between OPP exposure and the risk of T2DM have been extensively studied in the literature, including OPP-induced pancreatic β-cell dysfunction [48,49], dysregulation in hepatic gluconeogenesis [50,51], and an increase in oxidative stress and inflammation [52,53]. Cognitive impairment has been previously reported in both OPP exposure [54] and pre-diabetes/T2DM [55,56]. However, the biochemical mechanisms/pathways underlying this shared phenotype have thus far remained underexplored in the literature. Arachidonic acid, as mentioned earlier, serves as a critical precursor to a variety of biomolecules, including prostaglandins, thromboxanes, leukotrienes, lipoxins, and endocannabinoids. Notably, OPP exposure has been shown to disrupt the endocannabinoid pathway, which is critical for various neurological functions. In this study, we investigated the hippocampal alterations associated with OPP exposure and pre-diabetes that may contribute to cognitive impairment. The primary focus of this study was to identify potential mechanistic links between these conditions and the risk of neurodegenerative disease development. Furthermore, we sought to determine whether supplementation with AA, as a simple and inexpensive intervention, would help alleviate cognitive impairment by modulating the endocannabinoid system.

Insulin is not only responsible for maintaining glycemic control but also exerts critical functions in the brain via two major signaling pathways: the insulin-IRS-AKT pathway and the MAPK pathway [57,58,59,60]. Additionally, insulin receptors are highly enriched at synapses and are involved in enhancing neurite outgrowth, promoting dendritic outgrowth and development, and neurotransmitter release and uptake [61,62,63,64,65]. We observed that dermal exposure to chlorpyrifos was associated with a proteomic signature consistent with elevated hippocampal insulin resistance, indicated by the significant increase in levels of Rtn4 (reticulon 4) and Ldha (lactate dehydrogenase), alongside notable downregulation of Rpl26 (ribosomal protein L26), Atp5f1e (ATP synthase F1 subunit epsilon), and Ndufb11 (NADH dehydrogenase, ubiquinone, 1 beta subcomplex subunit 11). This predicted increase was consistent with the measured insulin resistance as displayed in Table 2.

Increased Ldha abundance may favor lactate production, although steady-state lactate is governed by cellular redox state and NAD(H) availability rather than enzyme abundance alone, and lactate also serves as neuronal fuel [66,67]. We therefore present the Ldha increase as a hypothesis linking it to lactate-associated signaling and cognitive impairment, to be tested by direct metabolic measurement. Additionally, increased lactate levels have previously been associated with diabetes-related cognitive impairment in rats [66]. Increased LDHA levels after chlorpyrifos exposure suggest a similar lactate-driven mechanism potentially contributing to cognitive impairment. Rtn4, also known as neurite outgrowth inhibitor (Nogo), serves as an inhibitor of neurite outgrowth in the central nervous system [68], with significantly increased levels being observed in both chlorpyrifos exposure and pre-diabetes compared to the control. Rtn4 is also known to act as a negative regulator of insulin signaling [69].

Rtn1 (Reticulon 1) and Rtn3 (Reticulon 3) were similarly upregulated following OPP exposure, consistent with heightened endoplasmic reticulum stress, neuritic dystrophy, and impaired synaptic plasticity, all of which are characteristic features of cognitive dysfunction [70,71]. Rtn1 functions as a mediator of ER-stress-induced neuronal death [72], with knockdown studies demonstrating increased neuroprotection [73]. Rtn3 instead accumulates in RTN3 immunoreactive dystrophic neurites (RIDNs), which are swollen dendrites or axons that represent a pathological hallmark of neurodegeneration [74]. Transgenic mice overexpressing Rtn3 spontaneously develop RIDNs in the hippocampal CA1 region, promoting neurodegeneration [75]. In contrast, Cdk5 (cell division protein kinase 5) is abundantly expressed in neurons and is responsible for maintaining proper neuronal migration [76,77], with deletions leading to increased neurodegeneration [78]. The downregulation of Cdk5, coupled with increases in Rtn1, Rtn3, and Rtn4, is consistent with a proteomic signature suggesting reduced neurite outgrowth, highlighting a potential cause of increased cognitive impairment following OPP exposure. Furthermore, the expression levels were similar in pre-diabetic rats, suggesting a common mechanism for cognitive impairment in both conditions.

OPPs have been known to disrupt mitochondrial function, leading to an increase in oxidative stress and an increased risk of various neurological disorders such as Alzheimer’s disease, Parkinson’s disease, and amyotrophic lateral sclerosis (ALS) [79]. Our data raises the possibility that mitochondrial perturbation extends beyond pancreatic β-cells to the hippocampus. The simultaneous downregulation of key mitochondrial proteins, including Atp5f1e, Ndufb11, Uqcrq, and Cox6a1, spans subunits of four major electron transport chain (ETC) complexes. Because changes in individual subunit abundance need not translate into altered complex activity or respiratory flux, this pattern is consistent with, but does not by itself establish, impaired mitochondrial respiration following chlorpyrifos exposure. The predicted activation of HIF-1α signaling after OPP exposure is likewise consistent with a shift away from oxidative phosphorylation: HIF-1α can redirect cellular metabolism by inducing pyruvate dehydrogenase kinase 1 (Pdk1), which phosphorylates and inactivates pyruvate dehydrogenase [80,81]. We additionally observed marked upregulation of Ppp3ca (calcineurin A alpha, catalytic subunit) and Ppp3r1 (calcineurin B, regulatory subunit), which together form calcineurin, a calcium/calmodulin-dependent phosphatase that serves as a primary sensor and transducer of calcium signals [82]. Concurrent upregulation of both subunits may indicate heightened calcium/calmodulin-dependent phosphatase signaling, although phosphatase activity was not measured directly here. Notably, our previous function work demonstrated hippocampal mitochondrial dysfunction, increased mitochondrial fission and oxidative stress, together with elevated HIF-1α signaling in the pre-diabetic rat model [83], providing functional context for the abundance level changes reported here.

A consistent increase in 14-3-3 proteins was observed in both chlorpyrifos exposure (CPY vs. CTRL) and pre-diabetes (PD vs. CTRL). Notably, Ywhaz (14-3-3 zeta), Ywhae (14-3-3 epsilon), and Ywhaq (14-3-3 theta) were all found to be upregulated in both conditions. Ywhae has previously been noted to inhibit insulin secretion by constraining mitochondrial respiration and ATP synthesis in pancreatic β-cells and may exert a similar effect on neuronal cells, with its localization in the mitochondria of hippocampal cells having been observed previously [84,85]. Additionally, increased levels of Ywhaz have previously been reported in diabetic db/db mice, with a similar change in chlorpyrifos exposure potentially contributing towards an increase in insulin resistance [84].

Dnaja3 (DnaJ homolog subfamily A member 3, mitochondrial) and Dnaja4 (DnaJ heat shock protein family member A4) are key chaperone proteins critical for cell function [86]. Dnaja3 acts as a mitochondrial co-chaperone and regulates the homeostasis of its client proteins [84]. In contrast, Dnaja4 is a cytosolic co-chaperone essential in ensuring proper protein folding [87]. Significant reductions in both proteins suggest altered chaperone activity common to chlorpyrifos exposure and pre-diabetes, and this alteration is consistent with heightened ER and mitochondrial stress.

This convergent proteomic signature points to a previously underappreciated molecular overlap between environmental neurotoxin exposure and metabolic disease. Both conditions shared an increase in the presynaptic proteins Snap25 (synaptosomal-associated protein 25) and Bsn (Bassoon) together with a decrease in Ptbp1 (polypyrimidine tract-binding protein 1), indicating a coordinated remodeling of synaptic machinery. This pattern is consistent with the progressive synaptic disruption described after OPP exposure, in which pre- and postsynaptic proteins are altered without initial neuronal death [88]. Previous studies have reported reduced levels of synaptophysin, snyapsin II, and PSD-95 in hippocampal dendritic zones, with compensatory upregulation of adhesion molecules following OPP exposure [89]. Because protein-abundance changes alone do not distinguish compensatory from maladaptive responses, the direction of these changes should be interpreted cautiously. The increase in Snap25, a core SNARE-complex component, and in Bsn, a presynaptic active-zone scaffold, may represent a compensatory attempt to maintain neurotransmitter-release capacity and active-zone organization under acetylcholinesterase inhibition (OPP) and insulin-signaling dysregulation (pre-diabetes) [90]. Equally, it could reflect maladaptive presynaptic remodeling, and the two possibilities cannot be separated without functional measures of synaptic transmission.

The shared decrease in Ptbp1 (Polypyrimidine tract-binding protein 1) in both conditions further represents a significant disruption of neuronal homeostasis. Ptbp1 functions as a regulator of splicing events essential for axon formation, synaptogenesis, and apoptosis [91,92]. Loss of ptpb1-dependent splicing control compromises the fine-tuning of neuronal architecture required for proper synaptic function and reduces synaptic plasticity when faced with metabolic and oxidative stress [93].

As discussed earlier, OPP exposure is known to directly contribute towards insulin resistance, a finding which was further supported by data generated in this study. The observed Brsk2 upregulation common to chlorpyrifos exposure and pre-diabetes provides further molecular evidence for this convergence. Brsk2 directly promotes hyperinsulinemia-coupled insulin resistance and β-cell exhaustion [94]. Similarly, dysregulation of Mecp2 expression is linked to chronic stress-induced symptoms and is associated with epigenetic mechanisms that impair both peripheral and central insulin signaling in Alzheimer’s Disease and diabetes [95].

Cognitive testing revealed a significant decrease in cognitive function following chlorpyrifos exposure, with the inherent cognitive impairment present in pre-diabetes being further exacerbated via continued chlorpyrifos exposure. Arachidonic acid supplementation proved effective in enhancing cognitive function in both cohorts (Table 2). Although arachidonic acid restored cognitive function, the underlying mechanism remains uncertain. We hypothesize that this recovery may be mediated by normalization of the dysregulated endocannabinoid pathway, though further investigation is required to confirm this. Notably, the restoration of cognitive function occurred despite only partial amelioration of mitochondrial and oxidative stress-related proteins, both of which remained active. The return of Atp5f1e, Uqcrq, Cox6a1, C0x7cl1, and Cox6b1 toward control levels after AA supplementation in the non-diabetic groups is consistent with a partial abundance-level normalization of the dysregulated electron transport chain, although other members, including Ndufb11, Atp4a, Atp5f1c, and Atp5f1b remained differentially abundant. The return of Rtn4 toward baseline may reflect the relief of its inhibitory effect on insulin signaling and neurite outgrowth, which could, in turn, contribute to improved cognitive function (Figure 3E). Similarly, the normalization of various neuronal development-related proteins, including Septin7, Dlg2, Ctnnb1, Nptn, Tanc2, Ezr, Tln2, Flna, Pfn1, and Erc2, is consistent with improved neuronal homeostasis in the pre-diabetic cohort. Ezr (Ezrin) supports neuronal migration, cell-shape signaling, and motility; Tanc2, a synaptic scaffolding protein, regulates mTOR signaling, synaptic assembly, and neuronal development, with reduced levels associated with impaired dendritic-spine and synapse formation [96]. Dlg1 is another synaptic scaffold protein essential for assembling multiprotein signal complexes at excitatory complexes [97]. The concurrent decrease in Ezr, Tanc2, and Dlg1 is thus consistent with potential deficits in synaptic structure and plasticity that could render the hippocampus more vulnerable to functional decline, and the increase in these proteins following AA supplementation further supports a partial, abundance-level recovery of synaptic and neuronal-development machinery.

## 5. Limitations

While AA supplementation demonstrated restorative effects on a subset of disrupted hippocampal proteins, many altered targets remained unaffected. This indicates that, although AA may help reverse the cognitive impairment associated with organophosphate exposure and pre-diabetes, comprehensive, multi-targeted strategies will likely be required to address the broader spectrum of dysregulated pathways. Moreover, AA is not a uniformly beneficial intervention: as the precursor of pro-inflammatory eicosanoids generated through the cyclooxygenase and lipoxygenase pathways, sustained AA supplementation could exert context-dependent or even detrimental effects, and the net benefit observed here should not be assumed to generalize across different doses, durations, or disease states. The balance between the pro- and anti-inflammatory mediators derived from AA therefore warrants dedicated evaluation before any translational application.

Several additional limitations should be considered when interpreting these findings. First, this was a pilot study with a limited sample size, focused specifically on shared molecular mechanisms in the hippocampus; accordingly, individual protein-level differences are best regarded as hypothesis-generating, and our interpretation emphasizes pathway-level convergence. Second, the proteomic data report relative protein abundance and therefore cannot, on their own, establish changes in enzyme activity, complex or receptor function, or metabolic flux; interpretations involving mitochondrial respiration, insulin signaling, and synaptic transmission consequently require functional confirmation—for example, respirometry or enzyme-activity assays for mitochondrial function, and direct measurement of hippocampal insulin signaling (e.g., IRS-1/Akt phosphorylation) to complement the systemic insulin-resistance phenotype captured here by HOMA-IR. Third, orthogonal validation was limited to targeted PRM for a subset of proteins, of which five reached statistical significance, while the remainder showed concordant but non-significant trends; proteins that were not validated are treated as preliminary. Fourth, cognitive function was assessed cross-sectionally, so the temporal trajectory of cognitive change—and the durability of the AA-associated recovery—could not be evaluated. Finally, because both conditions are known to exert effects across multiple brain regions [50,98,99], future studies should expand proteomic and functional analyses to other neural sites to better define the overlap and therapeutic targets. Additionally, given the early exploratory nature of this study, we sought to reduce biological variability imparted by cyclic fluctuations in female sex hormones, especially since sex hormones such as estrogen are known to modulate the endocannabinoid system, neuronal pathways, and neuroinflammatory responses that warrant examination in the future. The development of serum-based biomarkers likewise remains a key goal for early detection and risk stratification of diabetes following organophosphate exposure. Despite these limitations, the present study represents an important first step toward characterizing hippocampal changes associated with chronic organophosphate toxicity and pre-diabetes, particularly because the pathways and functions examined—insulin signaling, synaptic plasticity, dendritic growth and branching, and oxidative phosphorylation—are conserved across mammals, with many of the proteins studied having established human orthologs. Together, these findings underscore the need for more extensive research to delineate common neurobiological changes and advance targeted intervention strategies.

## 6. Conclusions

This study provides proteomic evidence that organophosphate pesticide exposure and pre-diabetes converge on shared molecular mechanisms underlying cognitive impairment in the hippocampus. The identification of 97 commonly altered proteins between the two conditions, with consistent directionality of change, suggests that environmental neurotoxicant exposure may accelerate or mimic the neurobiological trajectory of metabolic disease. The recurrence of proteomic changes implicating mitochondrial dysfunction, insulin resistance, and synaptic disruption across both conditions positions these pathways as candidate targets for further investigation in populations with overlapping environmental and metabolic risk factors. The partial efficacy of arachidonic acid supplementation in restoring cognitive performance, while leaving substantial pathway dysregulation unresolved, suggests that AA supplementation alone is insufficient to counteract the full breadth of molecular change, and multi-targeted approaches addressing metabolic and neurotoxic insults may be required. Given the limited sample size and hippocampal focus of this pilot study, these findings should be interpreted as hypothesis-generating, warranting validation across additional brain regions, larger cohorts, and complementary omics platforms. Ultimately, the shared proteomic signature identified here offers a molecular framework for understanding how chronic pesticide exposure may predispose individuals to cognitive decline through pathways that parallel pre-diabetic neurodegeneration.

## Figures and Tables

**Table 1:** Summary of sample sizes used for behavioral and LC-MS/MS analyses.

Code	Group	Overall Sample Size	LC-MS/MS Sample Size
CTRL	Control	10	5
CPY	CPY Exposure	10	5
CPY + AA	CPY Exposure followed by AA Treatment	10	5
PD	Pre-Diabetic Control	10	5
PD + CPY	Pre-Diabetic CPY Exposure	10	5
PD + CPY + AA	Pre-Diabetic CPY Exposure followed by AA Treatment	10	4

**Table 2:** Mean and standard error of the mean values for HOMA-IR, discrimination index (DI), and spontaneous alteration performance (SAP%) across each cohort.

Measure	Metric	CTRL	CPY	AA	PD	PD + CPY	PD + CPY + AA
**HOMA-IR**	Mean	0.4626	6.358 ^a^	2.011 ^b^	1.962	6.473 ^c^	2.152 ^d^
	Std. Err of Mean	0.05895	0.8053	0.2850	0.1026	0.3508	0.1448
**DI**	Mean	0.5299	0.05400 ^a^	0.4144 ^b^	0.08970 ^a^	−0.1623	0.5624 ^d^
	Std. Err of Mean	0.04171	0.06879	0.1227	0.1270	0.02903	0.1181
**SAP%**	Mean	75.01	35.08 ^a^	73.72 ^b^	50.68	46.40	69.65
	Std. Err of Mean	4.303	10.15	7.890	3.776	12.24	7.400

Statistical Significance assessed by two-way ANOVA followed by Sidak’s Multiple Comparisons test and denoted by: ^a^ Significantly different vs. CTRL, ^b^ Significantly different vs. CPY, ^c^ Significantly different vs. PD, and ^d^ Significantly different vs. PD/CPY.

**Table 3:** Summary of Differential Expression Analysis using Welch’s *t*-Test using *p*-value < 0.05 and |logFC| > 1 as significance thresholds.

Comparison	Differentially Expressed	Increased	Decreased
PD vs. CTRL	267	135	132
CPY vs. CTRL	212	113	99
CPY + AA vs. CPY	88	47	41
CPY + AA vs. CTRL	421	230	191
PD + CPY vs. PD	90	45	45
PD + CPY + AA vs. PD + CPY	122	53	69
PD + CPY + AA vs. PD	86	61	25

## Data Availability

The raw mass spectrometry data from this study are publicly available on the MASSIVE repository via the following ftp link: ftp://massive-ftp.ucsd.edu/v12/MSV000100539/ (Accessed on 14 June 2026).

## Supplementary Materials

The following supporting information can be downloaded at: https://www.mdpi.com/article/10.3390/biom16070952/s1. Figure S1: Principal component analyses for individual comparisons including CTRL vs. CPY, CPY + AA vs. CPY, CPY + AA vs. CTRL, PD + CPY vs. PD, PD + CPY + AA vs. PD + CPY, and PD + CPY + AA vs. PD. Volcano plots for CPY + AA vs. CTRL and PD + CPY + AA vs. PD. Figure S2: (A) Reactome enrichment analysis for CPY vs. CTRL displaying significant alteration in Rho GTPase signaling, cellular response to stress, aerobic respiration/electron transport chain, and apoptotic pathways. (B) PTM Enrichment showing that over 50% of the altered proteins were phosphoproteins, followed by acetylation and methylation. (C) KEGG enrichment showing significant change in proteins involved in various neurodegeneration pathways including amyotrophic lateral sclerosis, Alzheimer’s disease, Huntington’s disease, prion disease, and Parkinson’s disease. (D) Log2(fold change) of proteins involved in apoptosis showing significant increase in Ywhaz, Ywhae, Ywhaq, Dynl1, and Mapk1 and a significant decrease in Gsn, H1-4, H1-5, Diablol1. Figure S3: (A) Biological processes enrichment showing altered processes following further exposure of pre-diabetic rats to chlorpyrifos (PD + CPY vs. PD). Various pathways related to neuronal function were found to be dysregulated, including nervous system development, neuronal projection development, neuronal development, and neurogenesis. (B) KEGG enrichment highlighting significant alterations in IgSF CAM signaling and focal adhesion proteins. (C) Expression of proteins involved in neuron development that were disrupted following exposure of pre-diabetic rats to chlorpyrifos. (D) Majority of the altered proteins were found to be attenuated following AA supplementation (PD + CPY + AA vs. PD). Figure S4: Expression of proteins involved in neuronal function or development pathways that were altered following exposure of pre-diabetic rats to chlorpyrifos. Figure S5: Representative boxplots for peptides following similar trends in PRM and DDA for non-diabetic cohort. Figure S6: Representative boxplots for peptides following similar trends in PRM and DDA for the Pre-diabetic cohort. Table S1: Expression of DEPs across all comparisons. Comparisons may be identified via sheet names. Table S2: Expression of common DEPs between pre-diabetes and chlorpyrifos exposure. Table S3: Moderated t-statistic results using limma statistical analysis.

## Abbreviations

OPP: Organophosphate pesticide; CPY: Chlorpyrifos; PD: Pre-Diabetes; AA: Arachidonic Acid; CTRL: Control; DEP: Differentially expressed protein; LC-MS/MS: Liquid Chromatography-tandem mass spectrometry; LXR/RXR: Liver-X-Receptor/Retinoid-X-Receptor; DHCR24: 24-dehydrocholesterol reductase; LXA4: Lipoxins A4; LXB4: Lipoxins B4; 2-AG: 2-arachidonoylglycerol; AD: Alzheimer’s Disease; T2DM: Type 2 Diabetes Mellitus; TLR4: Toll-like receptor 4; VWF: von Willebrand factor; AAR: Alternate arm returns; DI: discrimination index; SDC: Sodium deoxycholate; DTT: Dithiothreitol; IAA: Iodoacetamide; ACN: Acetonitrile; FA: Formic Acid; MPA: Mobile phase A; MPB: Mobile phase B; AGC: Automated Gain Control; PCA: Principal component analysis; IPA: Ingenuity pathway analysis.
