# Supplementary material for: Hippocampal Mitochondrial Dysfunction and Synaptic Disruption Link Organophosphate Exposure to Pre-Diabetes: An LC-MS/MS-Based Proteomics Approach
Source: Biomolecules. 2026 Jun 26;16(7):952. doi: 10.3390/biom16070952 (PMC13406760; doi:10.3390/biom16070952)

Hippocampal Mitochondrial Dysfunction and Synaptic Disruption Link Organophosphate Exposure to Pre-Diabetes: An LC-MS/MS-Based Proteomics Approach

Vishal Sandilya [1], Rowan E. Arida [2,3], Sherifdeen Onigbinde [1], Sarah Sahioun [1], Favour Chukwubueze [1], Hadi Al Sheikh [1], Heba-Tallah Abd Elrahim Abd Elkader [4], Salwa A. Abuiessa [2], Mahmoud Agami [3], Mai M. Helmy [2], Ahmed El-Yazbi [2,3,*] and Yehia Mechref [1,*]

[1] Department of Chemistry and Biochemistry, Texas Tech University, Lubbock, TX 79409, USA;
vishal.sandilya@ttu.edu (V.S.); sonigbin@ttu.edu (S.O.); ssahioun@ttu.edu (S.S.); fchukwub@ttu.edu (F.C.); halsheik@ttu.edu (H.A.S.)
[2] Department of Pharmacology and Toxicology, Faculty of Pharmacy, Alexandria University, Alexandria 21521, Egypt; ressam@aiu.edu.eg (R.E.A.); salwa.aborajeh@alexu.edu.eg (S.A.A.); mai.helmy@alexu.edu.eg (M.M.H.)
[3] Faculty of Pharmacy and Research & Innovation Hub, Alamein International University, Alamein 51718, Egypt; mahmoud.a.agami@gmail.com
[4] Zoology, Biological, and Geological Sciences Department, Faculty of Education, Alexandria University, Alexandria 21526, Egypt; hebatallah@alexu.edu.eg
* Correspondence: ayazbi@aiu.edu.eg (A.E.-Y.); yehia.mechref@ttu.edu (Y.M.)

Figure S1: Principal component analyses for individual comparisons including CTRL vs. CPY, CPY + AA vs. CPY, CPY + AA vs. CTRL, PD + CPY vs. PD, PD + CPY + AA vs. PD + CPY, and PD + CPY + AA vs. PD. Volcano plots for CPY + AA vs. CTRL and PD + CPY + AA vs. PD.

Figure S2: (A) Reactome enrichment analysis for CPY vs. CTRL displaying significant alteration in Rho GTPase signaling, cellular response to stress, aerobic respiration/electron transport chain, and apoptotic pathways. (B) PTM Enrichment showing that over 50% of the altered proteins were phosphoproteins, followed by acetylation and methylation. (C) KEGG enrichment showing significant change in proteins involved in various neurodegeneration pathways including amyotrophic lateral sclerosis, Alzheimer's disease, Huntington's disease, prion disease, and Parkinson's disease. (D) Log2(fold change) of proteins involved in apoptosis showing significant increase in Ywhaz, Ywhae, Ywhaq, Dynl1, and Mapk1 and a significant decrease in Gsn, H1-4, H1-5, Diablol1.

Figure S3: (A) Biological processes enrichment showing altered processes following further exposure of pre-diabetic rats to chlorpyrifos (PD + CPY vs. PD). Various pathways related to neuronal function were found to be dysregulated, including nervous system development, neuronal projection development, neuronal development, and neurogenesis. (B) KEGG enrichment highlighting significant alterations in IgSF CAM signaling and focal adhesion proteins. (C) Expression of proteins involved in neuron development that were disrupted following exposure of pre-diabetic rats to chlorpyrifos. (D) Majority of the altered proteins were found to be attenuated following AA supplementation (PD + CPY + AA vs. PD).

Figure S4: Expression of proteins involved in neuronal function or development pathways that were altered following exposure of pre-diabetic rats to chlorpyrifos.

Figure S5: Representative boxplots for peptides following similar trends in PRM and DDA for non-diabetic cohort.

Figure S6: Representa-tive boxplots for peptides following similar trends in PRM and DDA for the Pre-diabetic cohort.

Table S1: Expression of DEPs across all comparisons. Comparisons may be identified via sheet names.

Table S2: Expression of common DEPs between pre-diabetes and chlorpyrifos exposure.

Table S3: Moderated t-statistic results using limma statistical analysis.

Supplementary Figure S1

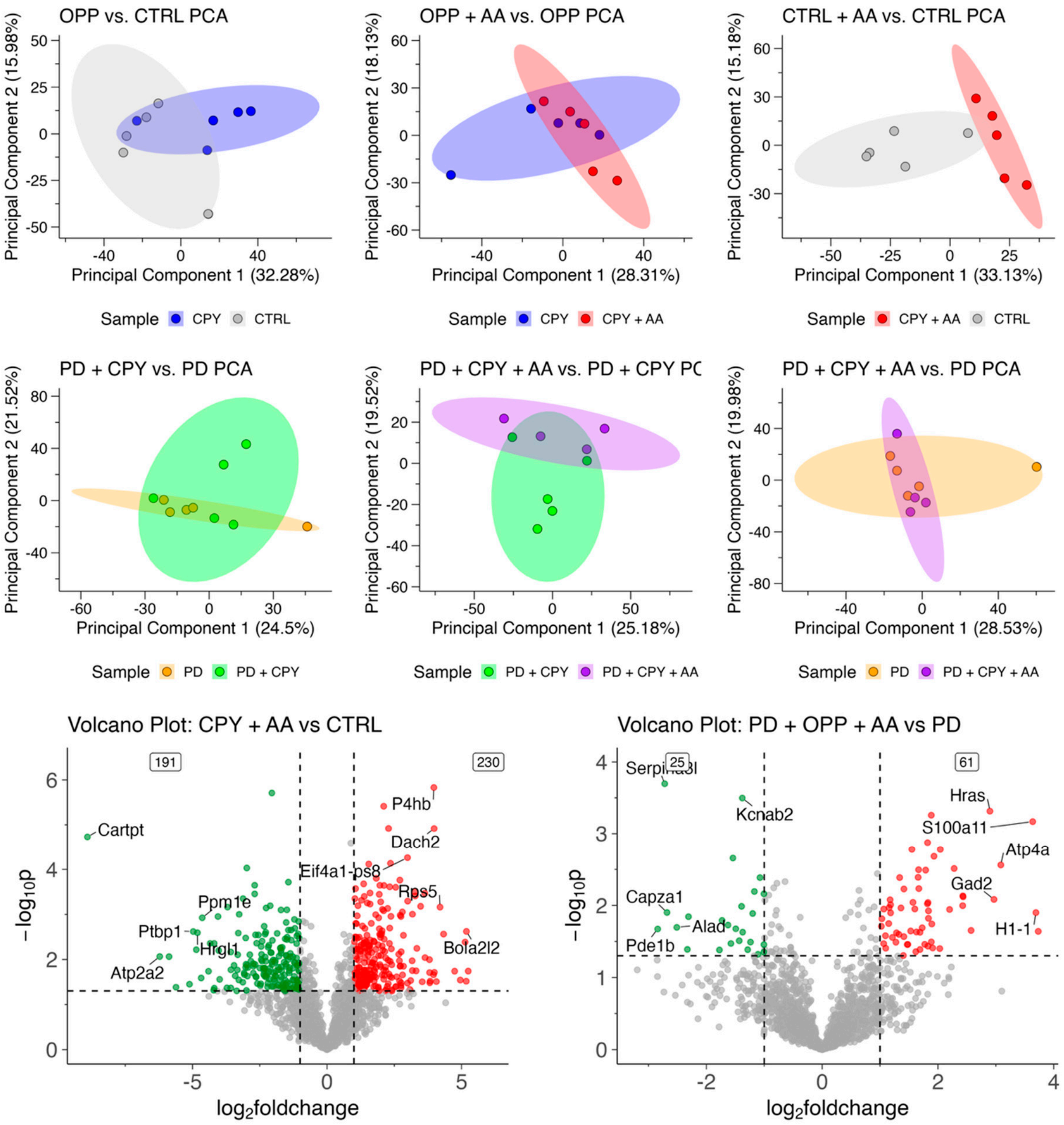

Supplementary Figure S2

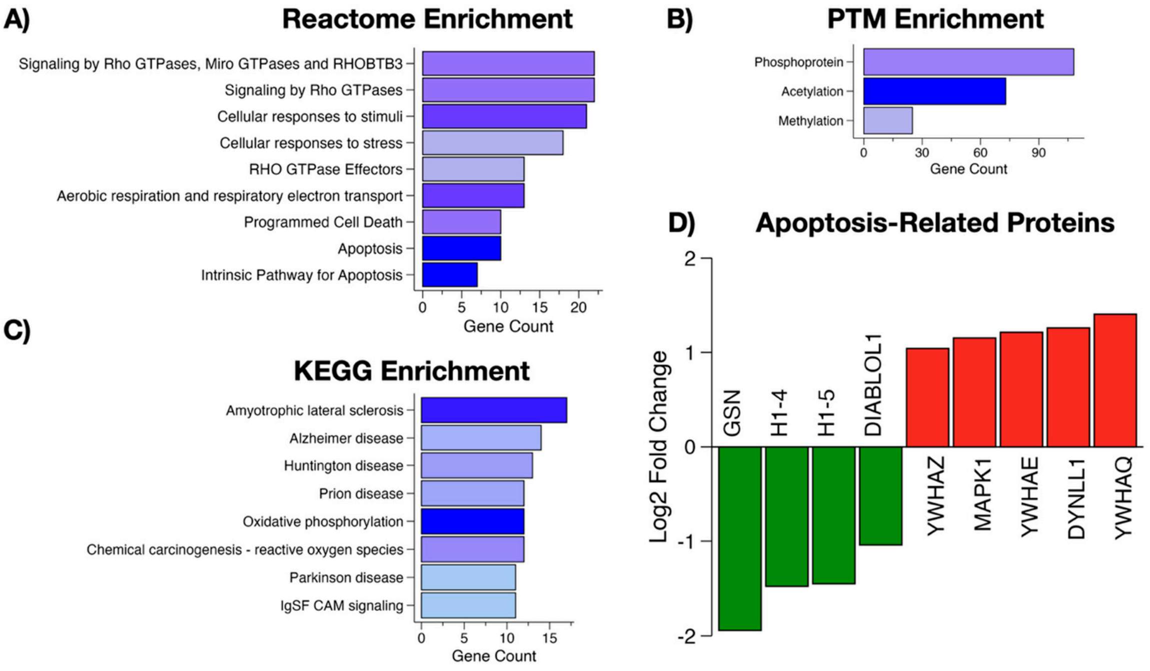

Supplementary Figure S3

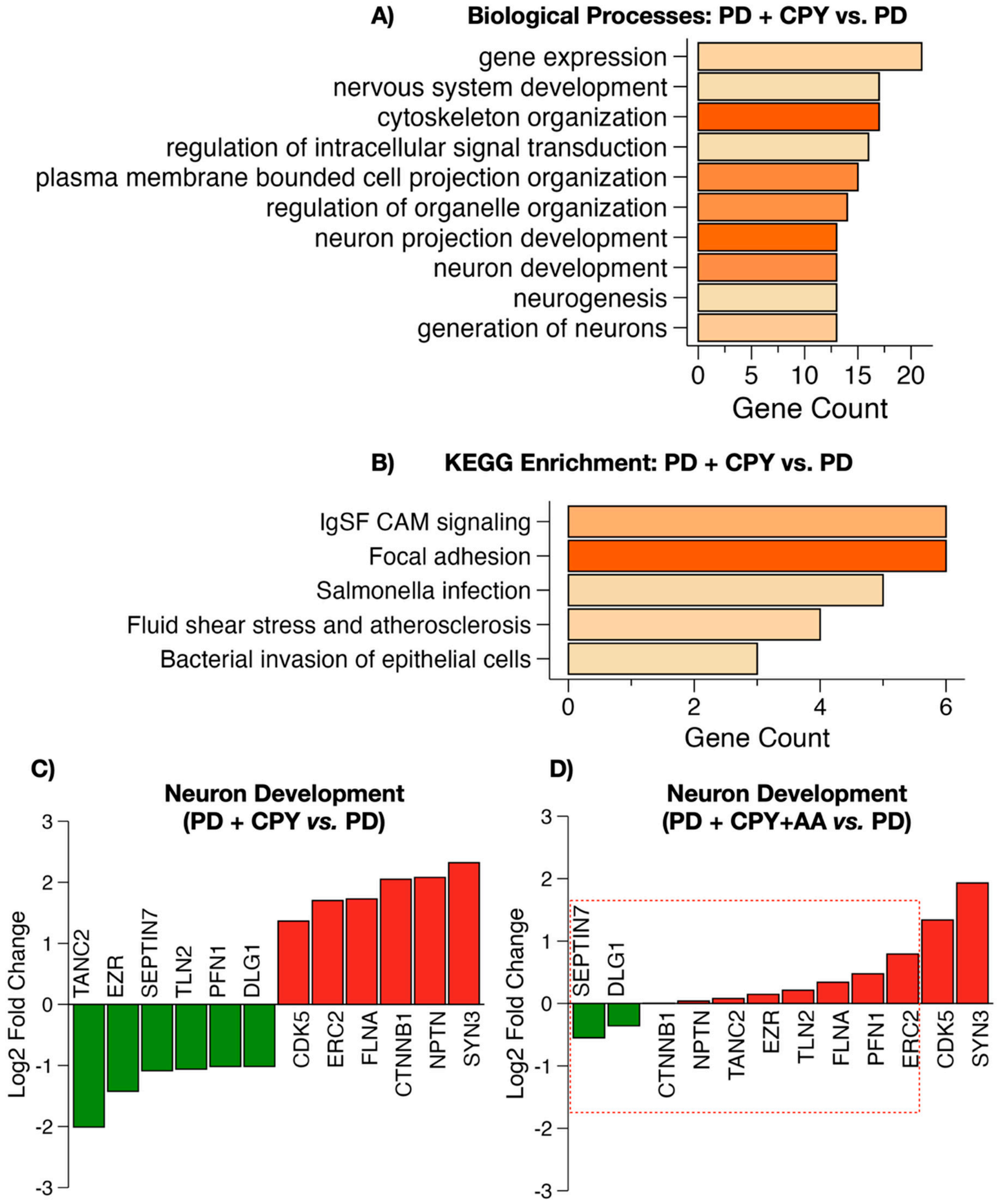

Supplementary Figure S4

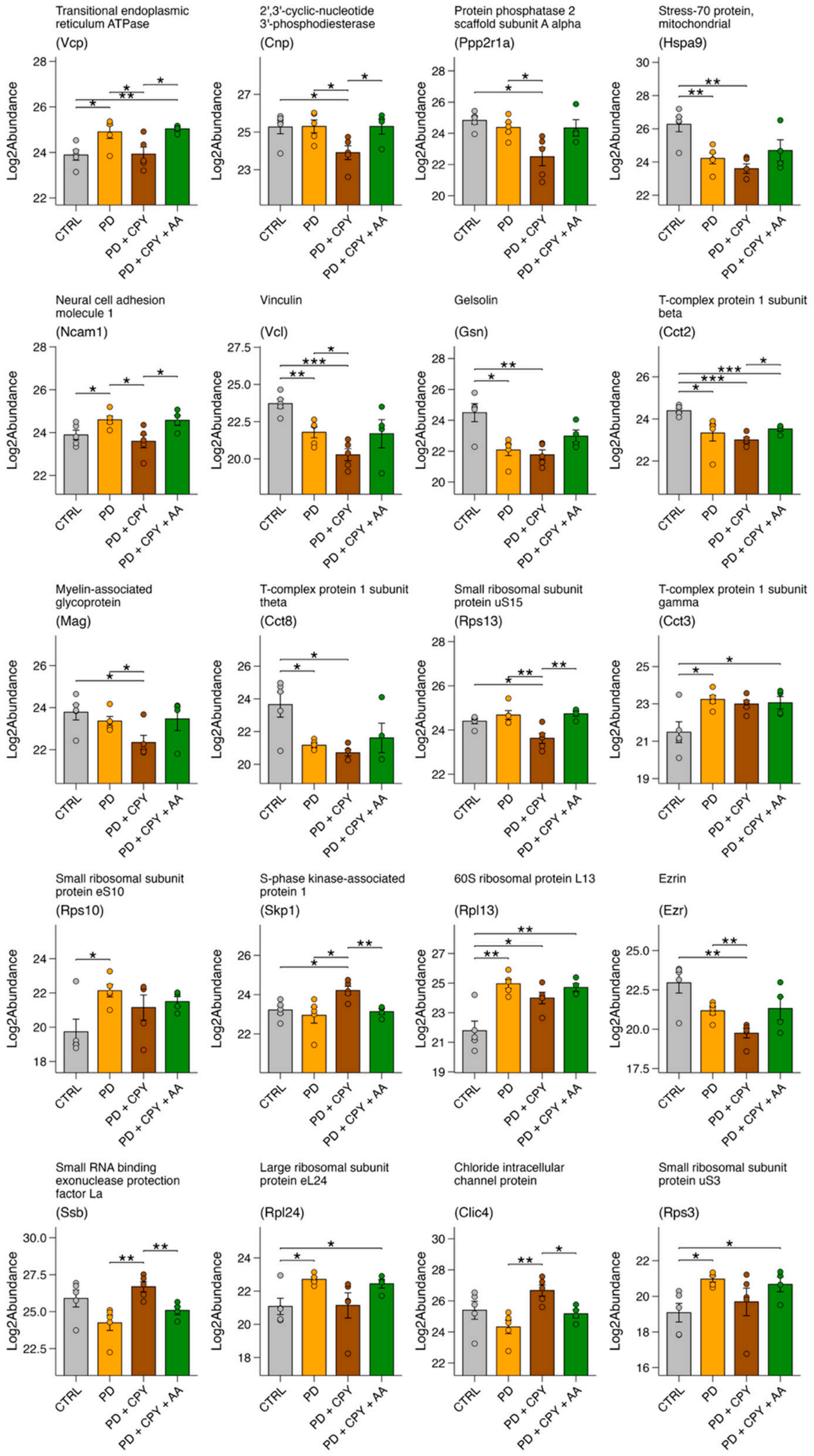

Supplementary Figure S5

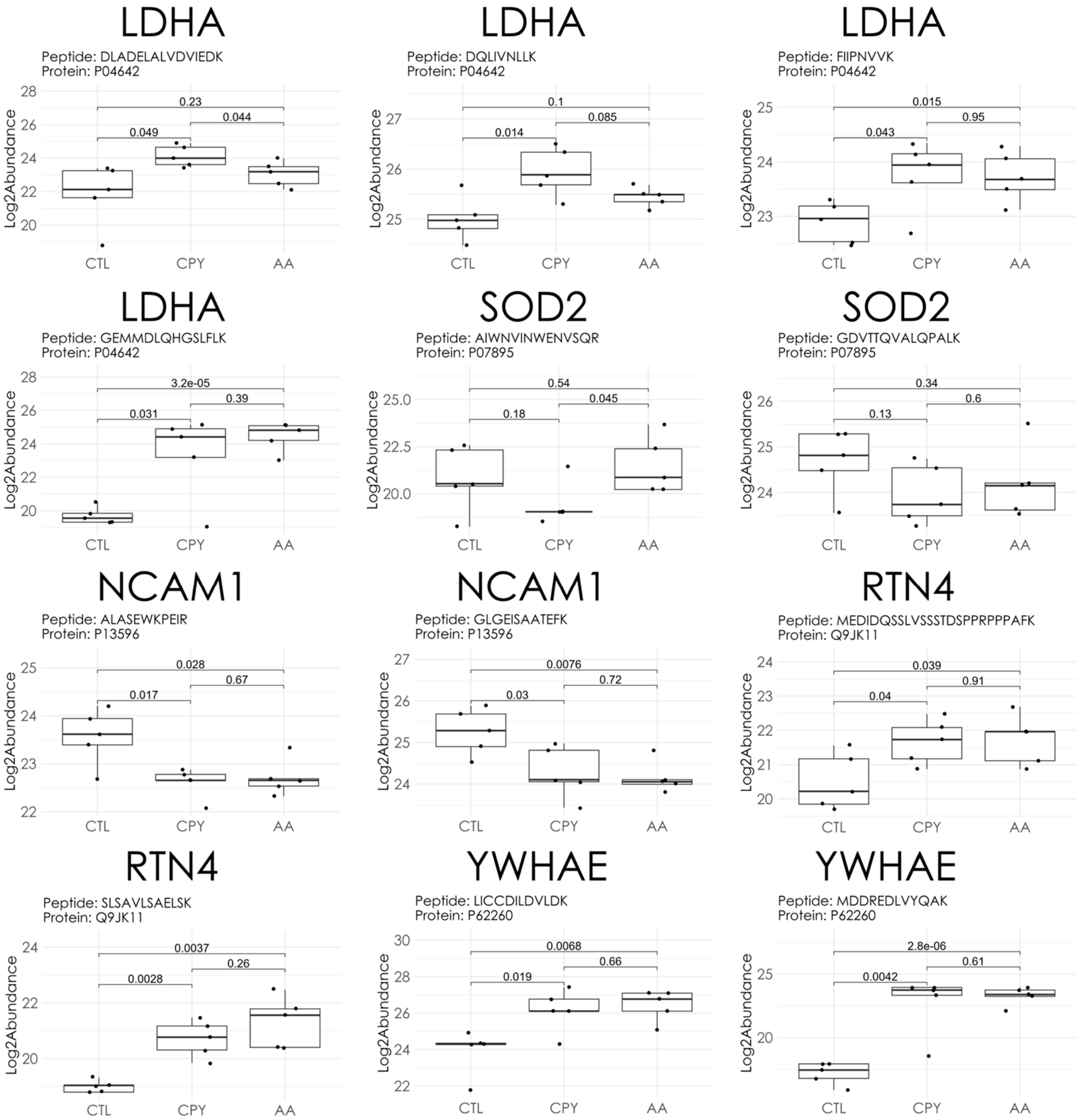

Supplementary Figure S6

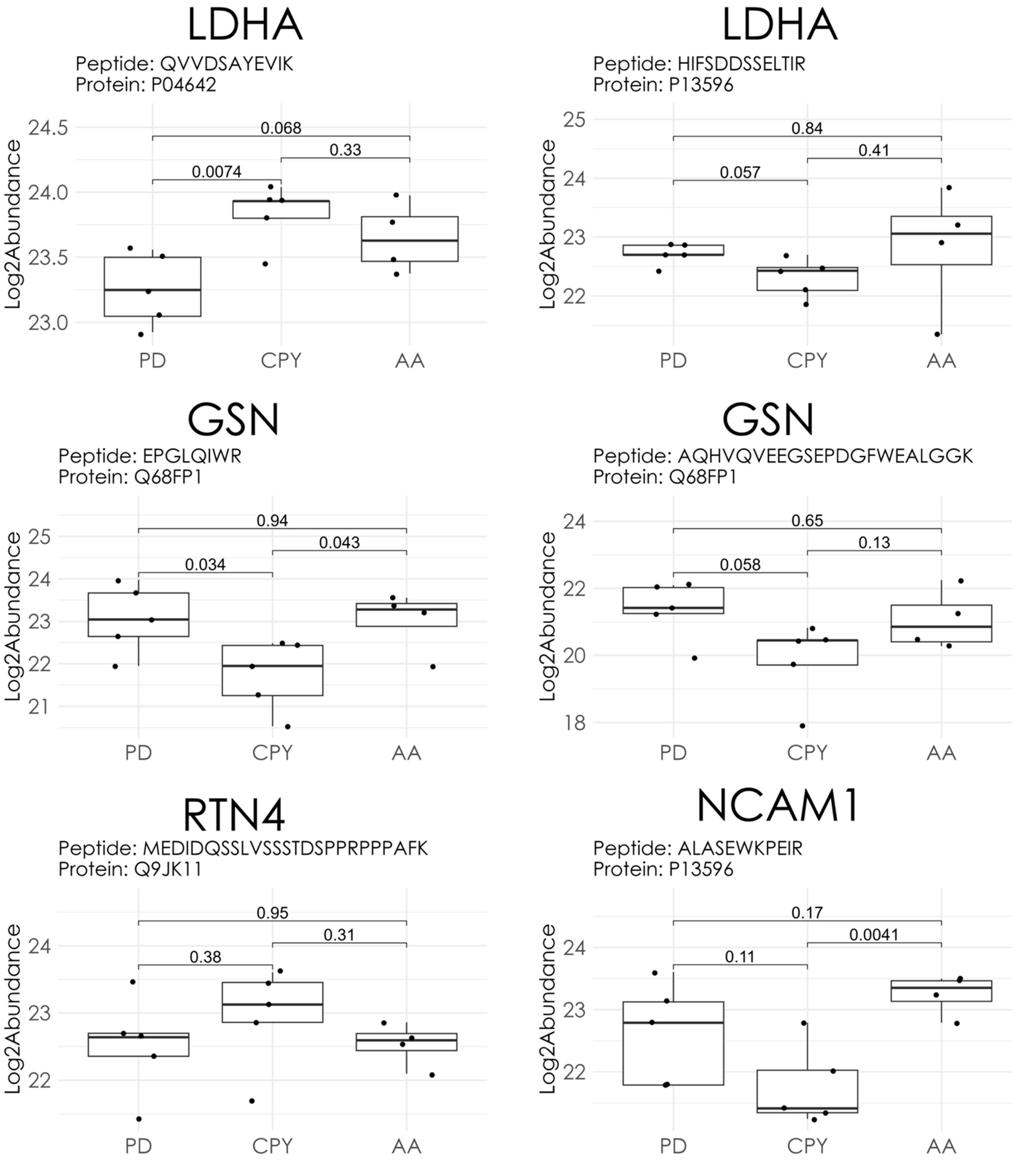

Supplement: Supplementary file 1 [file biomolecules-16-00952-s001.zip › SI Figures.pdf]
